# The association between the apolipoprotein A1/ high density lipoprotein -cholesterol and diabetes in Taiwan — a cross-sectional study

**DOI:** 10.1186/1472-6823-13-42

**Published:** 2013-10-07

**Authors:** Zhi-Hong Jian, Chia-Chi Lung, Pei-Chieh Ko, Yi-Hua Sun, Jing-Yang Huang, Chien-Chang Ho, Chia-Yo Ho, Yi-Chen Chiang, Chien-Jen Chen, Yung-Po Liaw

**Affiliations:** 1 Department of Public Health and Institute of Public Health, Chung Shan Medical University, Taichung City, Taiwan; 2 Department of Family and Community Medicine, Chung Shan Medical, University Hospital, Taichung City 40201, Taiwan; 3 Department of Dentistry, Chung Shan Medical University, Taichung 40201, Taiwan; 4 Department of Health and Leisure Management, Yuanpei University, Hsinchu, Taiwan; 5 Genomics Research Center, Academia Sinica, Taipei, Taiwan; 6 Graduate Institute of Epidemiology and Preventive Medicine, College of Public Health, National Taiwan University, Taipei, Taiwan

**Keywords:** Apolipoprotein A1, Apolipoprotein B, High density lipoprotein-cholesterol, Low density lipoprotein-cholesterol, Diabetes

## Abstract

**Background:**

Traditional lipid indices have been associated with type 2 diabetes, but it remains uncertain which lipid index is the best discriminator for diabetes. In this study, we aimed to assess lipoproteins, traditional lipid variables, and other variables to discover their association with diabetes in the Taiwanese population.

**Methods:**

Data from a nationwide cross-sectional population-based survey of 3087 men and 3373 women in 2002 were analyzed in this study. All participants were assessed for anthropometry, glycosylated hemoglobin, fasting sugar and lipid profiles with triglycerides, high density lipoprotein-cholesterol (HDL-C), low density lipoprotein-cholesterol (LDL-C), and apolipoprotein A1 (ApoA1) and B (ApoB). The ratio of LDL-C/HDL-C, ApoB/ApoA1, ApoB/LDL-C and ApoA1/HDL-C and other variables were analyzed to determine their potential roles in type 2 diabetes in the Taiwanese population. The Odds ratios (ORs) of the risk variables for diabetes were estimated using logistic regression and were adjusted for confounding factors.

**Results:**

The increased ratio of ApoA1/HDL-C was significantly associated with diabetes in men (top tertile vs. lowest: OR 2.98; 95% CI: 1.12 - 7.92; *P*-trend = 0.030) and women (top tertile vs. lowest: OR 2.15; 95% CI: 1.00 - 4.59; *P*-trend = 0.047). A modest increased diabetic risk was evident with ApoB/LDL-C in women (top tertile vs. lowest: OR 2.03; 95% CI: 1.07- 3.85; *P*-trend = 0.028), but not in men (top tertile v. lowest: OR 1.69; 95% CI: 0.79- 3.62; *P*-trend = 0.198).

**Conclusions:**

ApoA1/HDL-C had a significant linear association with diabetes in both sexes and was superior to other lipid and lipoprotein variables among the general Taiwanese population.

## Background

Diabetes mellitus is a growing health problem in Taiwan. According to data in the Taiwan’s National Health Insurance Research Database collected during 1999–2004, the age-standardized prevalence for Type 2 diabetes increased from 4.7% to 6.5% for men and from 5.3% to 6.6% for women [1]. Type 2 diabetes has been associated with an increased risk of cardiovascular disease [2], and the chronic comorbidity rates further increased after type 2 diabetes onset [3].

The identification of predisposing diabetes is essential for the successful implementation of primary prevention programs. The most commonly identified risk predictors were age, family history of diabetes, body mass index, abdominal obesity, hypertension, sex, fasting glucose level, physical inactivity and high density lipoprotein-cholesterol (HDL-C) [4]. Apolipoprotein A1(ApoA1) and apolipoprotein B (ApoB) are the major parts of the protein moieties of HDL and low density lipoprotein-cholesterol (LDL-C), respectively [5]. High concentrations of ApoA1 were found to be predictive of type-2 diabetes in Turkish adults [6]. The ratios of lipid and lipoprotein profiles, total cholesterol/HDL-C and ApoB/HDL-C, could be independently associated with later development of type 2 diabetes [7].

The reduction of HDL-C levels was associated with beta-cell dysfunction in subjects with impaired glucose tolerance [8]. ApoB showed positive correlation with insulin resistance, but ApoA1, ApoA1/ApoB, LDL-C/ApoB, and HDL-C/ApoA1 revealed inverse results in non-diabetic normoglycemic patients [9]. The ApoB/ApoA1 ratio and LDL-C were also reported to be positively correlated with insulin resistance in a Chinese population with abdominal obesity [10].

Despite the clinical importance of the above lipids, lipoproteins and their ratios, it is still unclear which index was the best discriminator for diabetes, and this factor has never before been investigated for type 2 diabetes. Because type-2 diabetes is highly prevalent among Taiwanese adults, we analyzed the levels of lipids, lipoproteins and other variables to determine their potential relationship with diabetes.

## Methods

### Health data source

This study is based on the Taiwanese Survey on Hypertension, Hyperglycemia, and Hyperlipidemia (TwSHHH) 2002 dataset. The survey was conducted and provided by the National Health Research Institutes and Bureau of Health Promotion, Taiwan. All participants provided written informed consent for participation. The TwSHHH database consists of anonymous secondary data and was released to the public for research purposes.

The TwSHHH was a nationwide cross-sectional survey to determine the prevalence of hypertension, hyperglycemia, and hyperlipidemia for non-institutionalized people in Taiwan. The TwSHHH sample was drawn from a subsample of the National Health Interview Survey (NHIS) conducted in 2001 with a multistage stratified systematic sampling scheme that used a probability method proportional to size. The details of this population have been previously described [11-13].

To summarize, 1648 communities were selected from all over Taiwan. Four households were randomly selected from each chosen community. Each mentally competent household member of 15 years or older was interviewed. The final sample size was 26,685 non-institutionalized residents from 6592 households. Because conducting a biomarker screening for all NHIS participants was not economically feasible, half of the selected NHIS townships/districts (824 communities with 3296 households and 10292 residents) in each stratum were randomly selected for the TwSHHH. Patients who had received lipid-lowering drugs were excluded from our study. Of these 10,292 participants, 6460 (62.8%) were finally enrolled and interviews, physical exams and lipid profile measurements were conducted. We analyzed data on anthropometry, LDL-C/HDL-C, ApoB/ApoA1, ApoB/LDL-C, ApoA1/HDL-C, and other variables to determine the potential associations with type 2 diabetes in the Taiwanese population.

### Data collection

Data on socio-demographic characteristics, including sex, age, physical activity, lifestyle, menopausal status, dietary habits, physician-diagnosed diseases, and medical history were collected using questionnaires completed during home visits. Exercise was defined as ‘yes’ for those who reported regular exercise at least 30 minutes per day, 3 times a week for more than 3 months, and ‘no’ for those who did not regularly exercise. Waist circumference and hip circumference were measured with an anthropometric tape to the nearest 0.1 cm, as recommended by the World Health Organization [14]. Waist to hip ratio (WHR) was calculated for further analysis.

### Blood pressure

Sitting blood pressure was measured by well-trained nurses during home visits. Two blood pressure readings were taken 10 seconds apart in the right arm after volunteers had sat down and rested for 5 to 10 minutes. A third blood pressure measurement was taken if the first two blood pressure readings varied by more than 10 mmHg. The average of the two closest readings was calculated and used for further analysis.

### Blood biochemistries

Volunteers were instructed to fast overnight for more than 12 hours before blood sampling. Venous blood samples were collected to determine ApoA1, ApoB, fasting glucose, glycosylated hemoglobin, triglyceride (TG), HDL-C, LDL-C, creatinine, and uric acid.

### Definitions of diabetic characteristics

Individuals with diabetes were defined as those with plasma fasting glucose ≥126 mg/dl, glycosylated hemoglobin ≥ 6.5% [15], and/or the current use of diabetic medication.

### Statistical analysis

All analyses were performed for separate sexes using the SAS software package, version 9.2 (SAS Institute, Cary, NC, USA). Data were expressed as mean and standard deviation. A *p* value <0.05 was considered statistically significant. Logistic regression models were used to estimate the odds ratio (OR) and 95% confidence interval (CI) for the association between variables and diabetes. Lipid, lipoprotein, anthropometry and age variables were included in the models as categorical variables, using tertiles based on participant distribution. Exercise habits, blood pressure, and fasting sugar, uric acid, and creatinine levels were also adjusted. To assess the linear trend in the ORs of lipid and lipoprotein profiles, the median value for each tertile was imported into the logistic regression model as an ordinal variable.

## Results

The number of participants with complete data for enrollment in all the statistical analyses was 3087 men and 3373 women. Of these, 267 men and 287 women had type-2 diabetes. The distribution of certain risk variables were stratified into tertiles with age, WHR, HDL-C, LDL-C, ApoA, and ApoB values from lowest to highest, and comparisons were made between men and women (Table 1). The comparative analysis indicated that men had significantly higher WHR, ApoB, systolic and diastolic blood pressure values, and uric acid levels, but lower HDL-C and ApoA1 levels, all demonstrating statistical significance.

**Table 1:** Demographic, anthropometric, clinical and laboratory characterization of study population

	**Men (n = 3,087)**			**Women (n = 3,373)**		***P*-value**
	**Mean**	**s.d**		**Mean**	**s.d**	
Age (y)			Age (y)			
Age < 45	30.47	8.56	Age < 45	31.01	8.34	0.051
45≦Age < 60	50.87	4.14	45≦Age < 60	51.05	4.16	0.372
Age≧60	69.93	4.9	Age≧60	69.38	7.32	0.167
WHR			WHR			
WHR < 0.84	0.80	0.06	WHR < 0.75	0.71	0.04	<0.0001
0.84≦WHR < 0.90	0.87	0.02	0.75≦WHR < 0.81	0.78	0.02	<0.0001
WHR≧0.90	0.94	0.05	WHR≧0.81	0.87	0.05	<0.0001
HDL-C (mg/dL)			HDL-C (mg/dL)			
HDL-C < 44	34.89	6.35	HDL-C < 52	43.25	6.67	<0.0001
44≦HDL-C < 56	49.47	3.42	52≦HDL-C < 64	57.26	3.42	<0.0001
HDL-C≧56	66.49	9.83	HDL-C≧64	74.07	9.21	<0.0001
LDL-C (mg/dL)			LDL-C (mg/dL)			
LDL-C < 103	87.81	10.94	LDL-C < 100	86.39	9.27	0.001
103≦LDL-C < 124	113.07	6.14	100≦LDL-C < 122	110.09	6.41	<0.0001
LDL-C≧124	144.88	19.34	LDL-C≧122	143.57	20.36	0.116
ApoA1 (mg/dL)			ApoA1 (mg/dL)			
ApoA1 < 126	114.24	9.25	ApoA1 < 139	126.64	10.33	<0.0001
126≦ApoA1 < 143	133.37	4.76	139≦ApoA1 < 158	147.69	5.29	<0.0001
ApoA1≧143	161.73	17.76	ApoA1≧158	177.71	18.61	<0.0001
ApoB (mg/dL)			ApoB (mg/dL)			
ApoB < 79	64.62	10.25	ApoB <72	60.43	8.19	<0.0001
79≦ApoB < 101	89.38	6.22	72≦ApoB < 93	81.38	5.93	<0.0001
ApoB≧101	119.00	16.76	ApoB≧93	113.16	18.76	<0.0001
SBP (mmHg)	119.03	16.40	SBP (mmHg)	112.52	18.95	<0.0001
DBP (mmHg)	77.70	10.98	DBP (mmHg)	72.30	10.88	<0.0001
FPG (mg/dL)	94.76	28.68	FPG (mg/dL)	93.82	28.25	0.206
HbA1c (%)	5.37	1.03	HbA1c (%)	5.34	1.05	0.154
Uric acid (mg/dL)	7.23	1.67	UA (mg/dL)	5.55	1.51	<0.0001
Creatinine (mg/dL)	1.03	0.28	CREA (mg/dL)	0.80	0.32	<0.0001

*Abbreviations*: *ApoA1* apolipoprotein A1; *ApoB* apolipoprotein B; *DBP* diastolic blood pressure; *FPG* fasting plasma glucose; *HbA1c* glycosylated hemoglobin; *HDL*-*C* high density lipoprotein-cholesterol; *LDL*-*C* low density lipoprotein-cholesterol; *SBP* systolic blood pressure; *WHR* waist-to-hip ratio.

Table 2 illustrates the ORs for diabetes in men according to various levels of LDL-C/HDL-C, ApoB/ApoA1, ApoB/LDL-C, and ApoA1/HDL-C. After multivariate adjustment by ApoA1, ApoB, HDL-C, LDL-C, TG, WHR, age, exercise, blood pressure, fasting plasma glucose, uric acid and creatinine, the ORs across tertiles of ApoA1/HDL-C were 1.00, 1.48, and 2.92 (*P*-trend =0.026) in Model 4. The adjusted ORs for LDL-C/HDL-C, ApoB/ApoA1, and ApoB/LDL-C were attenuated and became insignificant.

**Table 2:** Associations of ratio of lipids and lipoproteins with diabetes in men

		**Univariate**			Multivariate*		
		**OR**		**95% CI**	**OR**		**95% CI**
Model 1	LDL-C/HDL-C						
	Mid-tertile	1.52		1.06-2.20	1.16		0.58-2.32
	Top-tertile	3.33		2.41-4.61	0.90		0.34-2.41
	*P*-*trend*		<0.0001			0.856	
Model 2	ApoB/ApoA1						
	Mid-tertile	1.62		1.11-2.36	0.93		0.43-2.01
	Top-tertile	3.75		2.69-5.23	0.78		0.27-2.29
	*P*-*trend*		<0.0001			0.636	
Model 3	ApoB/LDL-C						
	Mid-tertile	1.99		1.36-2.92	1.53		0.79-2.98
	Top-tertile	4.33		3.06-6.12	1.65		0.80-3.44
	*P*-*trend*		<0.0001			0.203	
Model 4	ApoA1/HDL-C						
	Mid-tertile	1.01		0.71-1.43	1.48		0.74-2.96
	Top-tertile	2.25		1.66-3.03	2.92		1.15-7.40
	*P*-*trend*		<0.0001			0.026	

*Adjusted by ApoA1, ApoB, HDL-C, LDL-C, triglyceride, waist to hip ratio, age, exercise, blood pressure, fasting plasma glucose, uric acid and creatinine.

*Abbreviations*: *ApoA1* apolipoprotein A1; *ApoB* apolipoprotein B; *HDL*-*C* high density lipoprotein-cholesterol; *LDL*-*C* low density lipoprotein-cholesterol.

Unadjusted and adjusted ORs of LDL-C/HDL-C, ApoB/ApoA1, ApoB/LDL-C, and ApoA1/HDL-C tertiles for diabetes in women are presented in Table 3. After adjustment, the OR for top ApoB/LDL-C tertile was attenuated and significantly associated with diabetes (OR 1.91; 95% CI: 1.04- 3.50; *P*-trend = 0.036) in Model 7. The adjusted OR for the top ApoA1/HDL-C tertile remained significant and was independently associated with diabetes in Model 8, at OR of 2.24 (*P*-trend =0.028).

**Table 3:** Associations of ratio of lipids and lipoproteins with diabetes in women

		**Univariate**			Multivariate*		
		**OR**		**95% CI**	**OR**		**95% CI**
Model 5	LDL-C/HDL-C						
	Mid-tertile	1.72		1.21-2.46	0.89		0.51-1.56
	Top-tertile	3.57		2.59-4.92	0.64		0.29-1.41
	*P*-*trend*		<0.0001			0.272	
Model 6	ApoB/ApoA1						
	Mid-tertile	1.73		1.21-2.48	0.62		0.34-1.16
	Top-tertile	3.70		2.68-5.10	0.54		0.23-1.29
	*P*-*trend*		<0.0001			0.178	
Model 7	ApoB/LDL-C						
	Mid-tertile	1.99		1.37-2.88	1.40		0.81-2.42
	Top-tertile	4.33		3.10-6.06	1.91		1.04-3.50
	*P*-*trend*		<0.0001			0.036	
Model 8	ApoA1/HDL-C						
	Mid-tertile	1.14		0.81-1.58	1.26		0.72-2.22
	Top-tertile	2.23		1.66-2.99	2.24		1.09-4.62
	*P*-*trend*		<0.0001			0.028	

*Adjusted by ApoA1, ApoB, HDL-C, LDL-C, triglyceride, waist to hip ratio, age, exercise, blood pressure, fasting plasma glucose, uric acid and creatinine.

*Abbreviations*: *ApoA1* apolipoprotein A1; *ApoB* apolipoprotein B; *HDL-C* high density lipoprotein-cholesterol; *LDL-C* low density lipoprotein-cholesterol.

The ratios of LDL-C/HDL-C, ApoB/ApoA1, ApoB/LDL-C, and ApoA1/HDL-C were analyzed in the same model and the results are listed in Table 4. The top ApoA1/HDL-C tertile was significantly and independently associated with diabetes at ORs of 2.98 (*P*-trend = 0.030) for men and 2.15 (*P*-trend = 0.047) for women. The top tertile of ApoB/LDL-C was significantly associated with diabetes at an OR of 2.03 (*P*-trend = 0.028) in women, but not in men. No significant associations of ApoB/ApoA1 and LDL-C/HDL-C with diabetes in both sexes were observed.

**Table 4:** Multivariate logistic regression model for diabetes by sex

	**Men**		**Women**	
	**OR**	**95% CI**	**OR**	**95% CI**
ApoA1/HDL-C				
Mid-tertile	1.50	0.73-3.08	1.24	0.69-2.21
Top-tertile	2.98	1.12-7.92	2.15	1.00-4.59
*P*-*trend*	0.030		0.047	
ApoB/ApoA1				
Mid-tertile	0.96	0.43-2.15	0.65	0.33-1.24
Top-tertile	0.83	0.27-2.59	0.54	0.21-1.40
*P*-*trend*	0.731		0.213	
LDL-C/HDL-C				
Mid-tertile	1.08	0.52-2.23	0.96	0.54-1.72
Top-tertile	0.79	0.28-2.27	0.70	0.30-1.64
*P*-*trend*	0.678		0.431	
ApoB/LDL-C				
Mid-tertile	1.54	0.78-3.02	1.46	0.83-2.55
Top-tertile	1.69	0.79-3.62	2.03	1.07-3.85
*P*-*trend*	0.198		0.028	
ApoA1				
Mid-tertile	0.81	0.45-1.45	0.88	0.51-1.50
Top-tertile	0.87	0.41-1.85	1.52	0.78-2.96
HDL-C				
Mid-tertile	0.70	0.31-1.56	0.98	0.55-1.74
Top-tertile	1.12	0.36-3.50	0.81	0.33-1.95
ApoB				
Mid-tertile	0.70	0.28-1.71	1.53	0.73-3.20
Top-tertile	1.45	0.44-4.81	1.95	0.67-5.68
LDL-C				
Mid-tertile	1.45	0.66-3.20	0.98	0.50-1.92
Top-tertile	0.99	0.36-2.76	1.05	0.43-2.59
TG				
Mid-tertile	1.06	0.55-2.03	0.45	0.26-0.79
Top-tertile	1.02	0.49-2.11	0.55	0.30-0.99
WHR				
Mid-tertile	0.77	0.37-1.62	0.99	0.54-1.81
Top-tertile	1.00	0.48-2.09	1.54	0.83-2.87
Age < 45	-	-	-	-
45 ≤ Age < 60	2.15	1.19-3.88	1.11	0.65-1.87
Age ≥ 60	3.77	2.01-7.07	1.55	0.86-2.80
Exercise habits	0.78	0.47-1.28	1.20	0.78-1.85
SBP	1.00	0.98-1.02	1.01	0.99-1.02
DBP	0.99	0.96-1.02	0.98	0.96-1.01
FPG	1.13	1.11-1.15	1.11	1.09-1.12
UA	0.94	0.82-1.07	1.05	0.93-1.18
CREA	0.93	0.62-1.42	1.39	1.03-1.87

*Abbreviations*: *ApoA1* apolipoprotein A1; *ApoB* apolipoprotein B, *CREA* creatinine; *DBP* diastolic blood pressure; *FPG* fasting plasma glucose; *HDL*-*C* high density lipoprotein-cholesterol; *LDL*-*C* low density lipoprotein-cholesterol; *SBP* systolic blood pressure; *TG* triglyceride; *UA* uric acid; *WHR* waist-to-hip ratio.

## Discussion

Based on a thorough review of the literature, this study is the first to demonstrate that the ratio of ApoA1/HDL-C was the primary risk factor for diabetes in both sexes. Based on the TwSHHH study population, our findings also supported previous findings that traditional serum lipids are not strongly or consistently associated with diabetes. Notably, we observed that ApoA1/HDL-C was independently associated with diabetes in both sexes, and the serum ApoB/LDL-C ratio was only consistently associated with the presence of diabetes in women.

Disturbances in the production and clearance of plasma lipoproteins are commonly found in diabetic cases with metabolic abnormality. Diabetic dyslipidemia generally comprises postprandial lipidemia, high TG, reduced HDL-C, and low or relatively normal LDL-C [16], and the development of dyslipidemia may therefore be a signal of future diabetes onset. Although conventional lipid measures have been reported to be associated with incident type 2 diabetes, some debate still exits on the nature and extent of the association. Non-HDL-C was associated with incident type 2 diabetes and was superior to LDL-C or HDL-C at discriminating diabetes risk [17]. The association of ApoB with incident type 2 diabetes has been proven with improved risk prediction compared to LDL-C or HDL-C [18]. The ApoB/ApoA1 ratio is strongly associated with insulin resistance [19]. High ApoA1 levels independently predicted incident type-2 diabetes among a sample of Turkish participants and the top tertile of serum ApoA1 level nearly doubled the risk for incident diabetes when compared with the low tertile [6].

In diabetic dyslipidemia, LDL is converted to smaller, more atherogenic, ApoB containing lipoprotein particles termed small dense LDLs [20,21]. This feature was present in up to 50% of type 2 diabetic patients [22-24]. Dyslipidemia was also found in pre-diabetic patients with insulin resistance but with normal blood glucose indices [25]. Therefore, insulin resistance may increase the serum level of ApoB [26]. A previous study found that the ratio of ApoB/LDL-C was higher among diabetic cases than non-diabetic cases [27]. A low ratio of LDL-C/ApoB (reverse ApoB/LDL-C) has been reported to be the optimal indicator to detect dyslipidaemia among diabetic patients [28]. A Turkish population-based cohort study indicated the existence of greater independent associations of ApoB/LDL-C with future diabetic risk in women than in men [29]. For univariate analysis in this study, ApoA1/ HDL, ApoB/LDL, ApoB/ApoA1, and LDL/HDL were separately and significantly associated with diabetes. When they were separately put into the multivariate model for analyses, the ApoA1/HDL remained significant in both sexes, and the top tertile of ApoB/LDL was only significant in women. When a combination of ApoA1/ HDL, ApoB/LDL, ApoB/ApoA1 and LDL/HDL was used in the same model for analysis by sex, the lipid index of ApoA1/ HDL remained significant with a linear response in both sexes. Only the top ApoB/LDL tertile independently of other confounders conferred risk for diabetes at approximately 2-fold ORs in women but not in men. Further research is needed to explain the variance between sexes.

HDL participates in reverse cholesterol transport by removing excess cholesterol from peripheral tissues and delivering it to the liver for catabolism [30]. A reduced plasma HDL-C level is one of the key features of metabolic syndrome, a clinical entity known to be a predisposing factor for coronary heart disease and type 2 diabetes [31]. Therefore, HDL and HDL-C have anti-atherogenic properties [32]. In pre-diabetic patients, reduced HDL-C has been reported to be associated with progression to type 2 diabetes [8].

Because ApoA1 is the major protein component of HDL, low plasma ApoA1 levels have been associated with increased risk of coronary artery disease [33]. HDL is also considered a protective factor for diabetes. However, beneficial HDL can be degenerated to a dysfunctional HDL with pro-atherosclerotic and pro-inflammatory properties using oxidation [34] and glycation [35]. The lipid and apolipoprotein composition of HDL in the metabolic syndrome was obviously changed with impaired beneficial function and more readily detected the ApoA-I level [36]. The associations of increased ApoA-1/HDL-C ratio and abdominal obesity had also been reported in untreated newly diagnosed breast cancer patients [37].

Diabetic risk is related to central adiposity [38]. The consequence of abdominal obesity might be insulin resistance, which can lead to glucose intolerance and incident type 2 diabetes [39,40]. Body mass index (BMI), a general measure of obesity, has been reported to be closely associated with the risk of type 2 diabetes [41]. In the Taiwanese population, waist-to-hip ratio has been demonstrated to be a better predictor of risk of type 2 diabetes than BMI [42,43]. However, increased WHR was not significantly associated with diabetes in this study. More importantly, we found that high ApoA1/HDL-C ratio in serum was significantly associated with diabetes, and was superior to other variables, even after multiple-variable adjustments. In the top ApoA1/HDL-C tertile group, we found that HDL-C was the lowest among the tertiles for both sexes, but the ApoA1 concentrations were only slightly elevated. We suggest that the imbalance between ApoA1 and HDL-C serum levels was more critical in the pathogenesis of diabetes.

One advantage of this study was that selection biases could be reduced because the data from the national survey were representative of the general Taiwanese population. However, this study was not without limitations. The cross-sectional design of this study limited its ability to provide causal relationships between diabetes and various other lipid parameters. Nevertheless, this study was performed with a sizable number of participants who had not received lipid-lowering drugs, and therefore provides strong evidence of correlations between diabetes and various lipid parameters.

## Conclusions

In conclusion, ApoA1/HDL-C was highly associated with diabetes and was superior to ApoB/LDL-C, ApoA1, ApoB, LDL-C/HDL-C, ApoB/ApoA1, LDL-C, HDL-C, and TG among the general Taiwanese population. Whereas existing glucose-based diagnostic criteria remained unknown, an abnormal ApoA1/HDL-C in the Taiwanese population may indicate diabetes. A cohort study is needed to determine the role of ApoA1/HDL-C in the development of diabetes, as susceptible individuals are increasingly considered as candidates for appropriate interventions.

## Abbreviations

ApoA1: Apolipoprotein A1; ApoB: Apolipoprotein B; CI: Confidence interval; HDL-C: High density lipoprotein-cholesterol; LDL-C: Low density lipoprotein-cholesterol; NHIS: National Health Interview Survey; OR: Odds ratio; TG: Triglyceride; TwSHHH: The Taiwanese Survey on Prevalence of Hyperglycemia, Hyperlipidemia, and Hypertension; WHR: Waist to hip ratio.

## Competing interests

All the authors declare that they have no competing interests.

## Authors’ contributions

YPL designed the study. PCK, JYH and CYH extracted and analyzed data. ZHJ and CCL wrote the draft. YPL, YHS, YCC, CCH and CJC provided conceptual input and contributed to the final manuscript. All authors read and provided feedback on the draft versions of the article. All the authors have read and approved the final version.

## Pre-publication history

The pre-publication history for this paper can be accessed here:

http://www.biomedcentral.com/1472-6823/13/42/prepub
